# Peering through the mist: systematic review of what the chemistry of contaminants in electronic cigarettes tells us about health risks

**DOI:** 10.1186/1471-2458-14-18

**Published:** 2014-01-09

**Authors:** Igor Burstyn

**Affiliations:** 1 Department of Environmental and Occupational Health, School of Public Health, Drexel University, Nesbitt Hall, 3215 Market St. Floor 6, Office 614, Philadelphia, PA 19104, USA

**Keywords:** Vaping, e-cigarettes, Tobacco harm reduction, Risk assessment, Aerosol, Occupational exposure limit

## Abstract

**Background:**

Electronic cigarettes (e-cigarettes) are generally recognized as a safer alternative to combusted tobacco products, but there are conflicting claims about the degree to which these products warrant concern for the health of the vapers (e-cigarette users). This paper reviews available data on chemistry of aerosols and liquids of electronic cigarettes and compares modeled exposure of vapers with occupational safety standards.

**Methods:**

Both peer-reviewed and “grey” literature were accessed and more than 9,000 observations of highly variable quality were extracted. Comparisons to the most universally recognized workplace exposure standards, Threshold Limit Values (TLVs), were conducted under “worst case” assumptions about both chemical content of aerosol and liquids as well as behavior of vapers.

**Results:**

There was no evidence of potential for exposures of e-cigarette users to contaminants that are associated with risk to health at a level that would warrant attention if it were an involuntary workplace exposures. The vast majority of predicted exposures are < <1% of TLV. Predicted exposures to acrolein and formaldehyde are typically <5% TLV. Considering exposure to the aerosol as a mixture of contaminants did not indicate that exceeding half of TLV for mixtures was plausible. Only exposures to the declared major ingredients -- propylene glycol and glycerin -- warrant attention because of precautionary nature of TLVs for exposures to hydrocarbons with no established toxicity.

**Conclusions:**

Current state of knowledge about chemistry of liquids and aerosols associated with electronic cigarettes indicates that there is no evidence that vaping produces inhalable exposures to *contaminants* of the aerosol that would warrant health concerns by the standards that are used to ensure safety of workplaces. However, the aerosol generated during vaping as a whole (contaminants *plus declared ingredients*) creates personal exposures that would justify surveillance of health among exposed persons in conjunction with investigation of means to keep any adverse health effects as low as reasonably achievable. Exposures of bystanders are likely to be orders of magnitude less, and thus pose no apparent concern.

## Background

Electronic cigarettes (also known as e-cigarettes) are generally recognized as a safer alternative to combusted tobacco products (reviewed in [1]), but there are conflicting claims about the degree to which these products warrant concern for the health of the vapers (e-cigarette users). A vaper inhales aerosol generated during heating of liquid contained in the e-cigarette. The technology and patterns of use are summarized by Etter [1], though there is doubt about how current, complete and accurate this information is. Rather conclusive evidence has been amassed to date on comparison of the chemistry of aerosol generated by electronic cigarettes to cigarette smoke [2-8]. However, it is meaningful to consider the question of whether aerosol generated by electronic cigarettes would warrant health concerns on its own, in part because vapers will include persons who would not have been smokers and for whom the question of harm reduction from smoking is therefore not relevant, and perhaps more importantly, simply because there is value in minimizing the harm of those practicing harm reduction.

One way of approaching risk evaluation in this setting is to rely on the practice, common in occupational hygiene, of relating the chemistry of industrial processes and the emissions they generate to the potential worst case of personal exposure and then drawing conclusions about whether there would be interventions in an occupational setting based on comparison to occupational exposure limits, which are designed to ensure safety of unintentionally exposed individuals. In that context, exposed individuals are assumed to be adults, and this assumption appears to be suitable for the intended consumers of electronic cigarettes. “Worst case” refers to the maximum personal exposure that can be achieved given what is known about the process that generates contaminated atmosphere (in the context of airborne exposure considered here) and the pattern of interaction with the contaminated atmosphere. It must be noted that harm reduction notions are embedded in this approach since it recognizes that while elimination of the exposure may be both impossible and undesirable, there nonetheless exists a level of exposure that is associated with negligible risks. To date, a comprehensive review of the chemistry of electronic cigarettes and the aerosols they generate has not been conducted, depriving the public of the important element of a risk-assessment process that is mandatory for environmental and occupational health policy-making.

The present work considers both the contaminants present in liquids and aerosols as well as the declared ingredients in the liquids. The distinction between exposure to declared ingredients and contaminants of a consumer product is important in the context of comparison to occupational or environmental exposure standards. Occupational exposure limits are developed for unintentional exposures that a person does not elect to experience. For example, being a bread baker is a choice that does not involve election to be exposed to substances that cause asthma that are part of the flour dust (most commonly, wheat antigens and fungal enzymes). Therefore, suitable occupational exposure limits are created to attempt to protect individuals from such risk on the job, with no presumption of “assumed risk” inherent in the occupation. Likewise, special regulations are in effect to protect persons from unintentional exposure to nicotine in workplaces (http://www.cdc.gov/niosh/docs/81-123/pdfs/0446.pdf; accessed July 12, 2013), because in environments where such exposures are possible, it is reasonable to protect individuals who do not wish to experience its effects. In other words, occupational exposure limits are based on protecting people from involuntary and unwanted exposures, and thus can be seen as more stringent than the standards that might be used for hazards that people intentionally choose to accept.

By contrast, a person who elects to lawfully consume a substance is subject to different risk tolerance, as is demonstrated in the case of nicotine by the fact that legally sold cigarettes deliver doses of nicotine that exceed occupational exposure limits [9]: daily intake of 20 mg of nicotine, assuming nearly 100% absorption in the lungs and inhalation of 4 m^3^ of air, corresponds to roughly 10 times the occupational exposure limit of 0.5 mg/m^3^ atmosphere over 8 hours [10]. Thus, whereas there is a clear case for applicability of occupational exposure limits to contaminants in a consumer product (e.g. aerosol of electronic cigarettes), there is no corresponding case for applying occupational exposure limits to declared ingredients desired by the consumer in a lawful product (e.g. nicotine in the aerosol of an electronic cigarette). Clearly, some limits must be set for voluntary exposure to compounds that are known to be a danger at plausible doses (e.g. limits on blood alcohol level while driving), but the regulatory framework should reflect whether the dosage is intentionally determined and whether the risk is assumed by the consumer. In the case of nicotine in electronic cigarettes, if the main reason the products are consumed is as an alternative source of nicotine compared to smoking, then the only relevant question is whether undesirable exposures that accompany nicotine present health risks, and the analogy with occupational exposures holds. In such cases it appears permissible to allow at least as much exposure to nicotine as from smoking before admitting to existence of new risk. It is expected that nicotine dosage will not increase in switching from smoking to electronic cigarettes because there is good evidence that consumers adjust consumption to obtain their desired or usual dose of nicotine [11]. The situation is different for the vapers who want to use electronic cigarettes without nicotine and who would otherwise not have consumed nicotine. For these individuals, it is defensible to consider total exposure, including that from any nicotine contamination, in comparison to occupational exposure limits. In consideration of vapers who would never have smoked or would have quit entirely, it must be remembered that the exposure is still voluntary and intentional, and comparison to occupational exposure limits is legitimate only for those compounds that the consumer does not elect to inhale.

The specific aims of this review were to:

1. Synthesize evidence on the chemistry of liquids and aerosols of electronic cigarettes, with particular emphasis on the contaminants.

2. Evaluate the quality of research on the chemistry of liquids and aerosols produced by electronic cigarettes.

3. Estimate potential exposures from aerosols produced by electronic cigarettes and compare those potential exposures to occupational exposure standards.

## Methods

### Literature search

Articles published in peer-reviewed journals were retrieved from *PubMed* (http://www.ncbi.nlm.nih.gov/pubmed/) available as of July 2013 using combinations of the following keywords: “electronic cigarettes”, “e-cigarettes”, “smoking alternatives”, “chemicals”, “risks”, “electronic cigarette vapor”, “aerosol”, “ingredients”, “e-cigarette liquid”, “e-cig composition”, “e-cig chemicals”, “e-cig chemical composition”, “e-juice electronic cigarette”, “electronic cigarette gas”, “electronic cigars”. In addition, references of the retrieved articles were examined to identify further relevant articles, with particular attention paid to non-peer reviewed reports and conference presentations. Unpublished results obtained through personal communications were also reviewed. The Consumer Advocates for Smoke-free Alternatives Association (CASAA) was asked to review the retrieved bibliography to identify any reports or articles that were missed. The papers and reports were retained for analysis if they reported on the chemistry of e-cigarette liquids or aerosols. No explicit quality control criteria were applied in selection of literature for examination, except that secondary reporting of analytical results was not used. Where substantial methodological problems that precluded interpretation of analytical results were noted, these are described below. For each article that contained relevant analytical results, the compounds quantified, limits of detection, and analytical results were summarized in a spreadsheet. Wherever possible, individual analytical results (rather than averages) were recorded (see Additional file 1). Data contained in Additional file 1 is not fully summarized in the current report but can be used to investigate a variety of specific questions that may interest the reader. Each entry in Additional file 1 is identified by a *Reference Manage ID* that is linked to source materials in a list in Additional file 2 (linked via *RefID*); copies of all original materials can be requested.

### Comparison of observed concentrations in aerosol to occupational exposure limits

For articles that reported mass or concentration of specific compounds in the aerosol (generated by smoking machines or from volunteer vapers), measurements of compounds were converted to concentrations in the “personal breathing zone”,^a^ which can be compared to occupational exposure limits (OELs). The 2013 Threshold Limit Values (TLVs) [10] were used as OELs because they are the most up to date and are most widely recognized internationally when local jurisdictions do not establish their own regulations (see http://www.ilo.org/safework/info/publications/WCMS_113329/lang--en/index.htm; accessed July 3, 2013). TLVs are more protective that of US Occupation Safety and Health Administration’s Permissible Exposure Limits because TLVs are much more often updated with current knowledge. However, all OELs generally agree with each other because they are based on the same body of knowledge. TLVs (and all other OELs) aim to define environmental conditions to which nearly all persons can be exposed to all day over many years without experiencing adverse health effects. Whenever there was an uncertainty in how to perform the calculation, a “worst case” scenario was used, as is the standard practice in occupational hygiene, where the initial aim is to recognize potential for hazardous exposures and to err on the side of caution. The following assumptions were made to enable the calculations that approximate the worst-case personal exposure of a vaper (Equation 1):

(1) $$\begin{array}{ll} \left[\mathrm{mg}/m^{3}\right]= & \mathrm{mg}/\text{puff}\times \text{puffs}/\left(8\;\mathrm{hr}\;\text{day}\right)\\ & \times 1/\left(m^{3}\;\text{air}\;\text{inhaled}\;\mathrm{in}\;8\;\mathrm{hr}\right) \end{array}$$

1. Air the vaper breathes consists of a small volume of aerosol generated by e-cigarettes that contains a specific chemical plus pristine air;

2. The volume of aerosols inhaled from e-cigarettes is small compared to total volume of air inhaled;

3. The period of exposure to the aerosol considered was 8 hours for comparability to the standard working shift for which TLVs were developed (this does not mean only 8 hours worth of vaping was considered but, rather, a day's worth of exposure was modeled as being concentrated into just 8 hours);

4. Consumption of 150 puffs in 8 hours (an upper estimate based on a rough estimate of 150 puffs by a typical vaper in a day [1]) was assumed. (Note that if vaping over 16 hours “day” was considered then air into which contaminants from vaping are diluted into would have to increase by a factor of 2, thereby lowering estimated exposure; thus, the adopted approach is entirely still in line with “worst case” assessment);

5. Breathing rate is 8 liters per minute [12,13];

6. Each puff contains the same quantity of compounds studied.

The only exception to this methodology was when assessing a study of aerosol emitted by 5 vapers in a 60 m^3^ room over 5 hours that seemed to be a sufficient approximation of worst-case “bystander” exposure [6]. All calculated concentrations were expressed as the most stringent (lowest) TLV for a specific compound (i.e. assuming the most toxic form if analytical report is ambiguous) and expressed as “percent of TLV”. Considering that all the above calculations are approximate and reflecting that exposures in occupational and general environment can easily vary by a factor of 10 around the mean, we added a 10-fold safety factor to the “percent of TLV” calculation. This safety factor accounts for considerable uncertainty about the actual number and volume of puffs since the number of puffs is hard to estimate accurately with reports as high as 700 puffs per day [14]. Details of all calculations are provided in an Excel spreadsheet (see Additional file 3).

No systematic attempt was made to convert the content of the studied liquids into potential exposures because sufficient information was available on the chemistry of aerosols to use those studies rather than making the necessary simplifying assumptions to do the conversion. However, where such calculations were performed in the original research, the following approach was used: under the (probably false – see the literature on formation of carbonyl compounds below) assumption of no chemical reaction to generate novel ingredients, composition of liquids can be used to estimate potential for exposure if it can be established how much volume of liquid is consumed in given 8 hours, following an algorithm analogous to the one described above for the aerosols (Equation 2):

(2) $$\begin{array}{ll} \left[\mathrm{mg}/m^{3}\right]= & \mathrm{mg}/\left(\mathrm{mL}\;\text{liquid}\right)\times \left(\mathrm{mL}\;\text{liquid}\right)/\text{puff}\\ & \times \text{puffs}/\left(8\;\mathrm{hr}\;\text{day}\right)\\ & \times 1/\left(m^{3}\;\text{air}\;\text{inhaled}\;\mathrm{in}\;8\;\mathrm{hr}\right) \end{array}$$

Comparison to cigarette smoke was not performed here because the fact that e-cigarette aerosol is at least orders of magnitude less contaminated by toxic compounds is uncontroversial [2-8].

The study adhered to the PRISMA guidelines for systematic reviews (http://www.prisma-statement.org/).

## Results and discussion

### General comments on methods

In excess of 9,000 determinations of single chemicals (and rarely, mixtures) were reported in reviewed articles and reports, typically with multiple compounds per electronic cigarette tested [2-8,15-43]. Although the quality of reports is highly variable, if one assumes that each report contains some information, this asserts that quite a bit is known about composition of e-cigarette liquids and aerosols. The only report that was excluded from consideration was work of McAuley *et al.*[24] because of clear evidence of cross-contamination – admitted to by the authors – with cigarette smoke and, possibly, reagents. The results pertaining to non-detection of tobacco-specific nitrosamines (TSNAs) are potentially trustworthy, but those related to polycyclic aromatic hydrocarbons (PAH) are not since it is incredible that cigarette smoke would contain fewer PAHs, which arise from incomplete combustion of organic matter, than aerosol of e-cigarettes that do not burn organic matter [24]. In fairness to the authors of that study, similar problems may have occurred in other studies but were simply not reported, but it is impossible to include a paper in a review once it is known for certain that its quantitative results are not trustworthy. When in doubt, we erred on the side of trusting that proper quality controls were in place, a practice that is likely to increase appearance of atypical or erroneous results in this review. From this perspective, assessment of concordance among independent reports gains higher importance than usual since it is unlikely that two experiments would be flawed in the same exact manner (though of course this cannot be assured).

It was judged that the simplest form of publication bias – disappearance of an entire formal study from the available literature – was unlikely given the exhaustive search strategy and the contested nature of the research question. It is clearly the case that only a portion of all industry technical reports were available for public access, so it is possible that those with more problematic results were systematically suppressed, though there is no evidence to support this speculation. No formal attempt was made to ascertain publication bias *in situ* though it is apparent that anomalous results do gain prominence in typical reviews of the literature: diethylene glycol [44,45] detected at non-dangerous levels (see details below) in one test of 18 of early-technology products by the US Food and Drugs Administration (FDA) [23] and one outlier in measurement of formaldehyde content of exhaled air [4] and aldehydes in aerosol generated from one e-cigarette in Japan [38]. It must be emphasized that the alarmist report of aldehydes in experiments presented in [38] is based on the concentration in generated aerosol rather than air inhaled by the vaper over prolonged period of time (since vapers do not inhale only aerosol). Thus, results reported in [38] cannot be the basis of any claims about health risk, a fallacy committed both by the authors themselves and commentators on this work [45].

**Figure 1:**
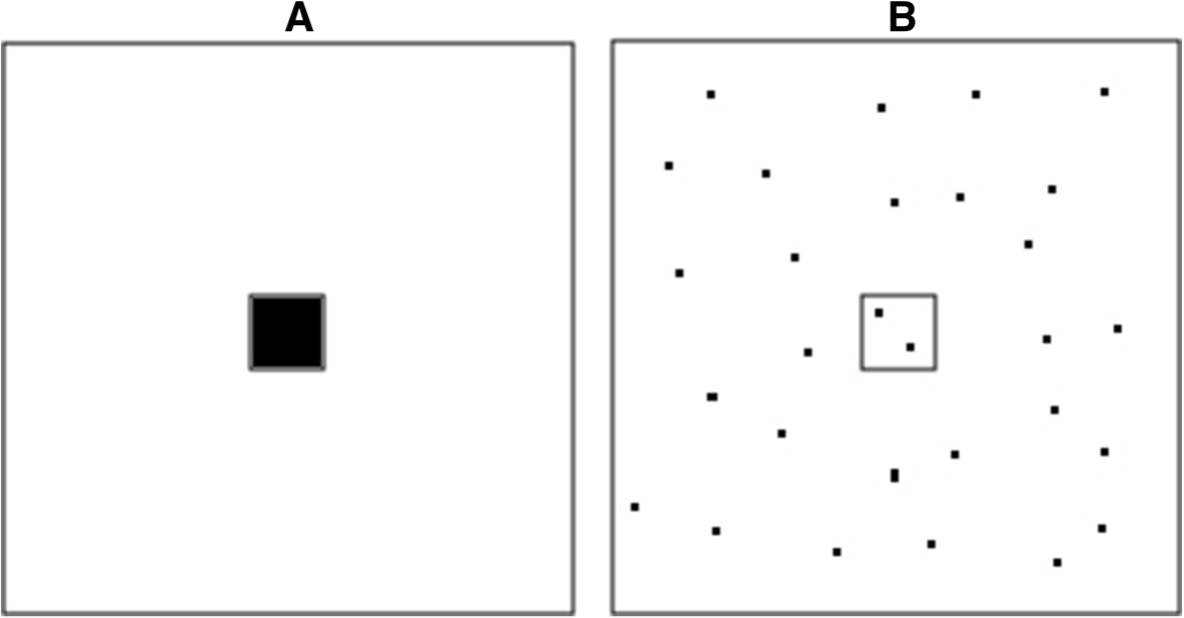
**Illustrating the difference between concentrations in the aerosol generated by vaping and inhaled air in a day. ***Panel ****A ***shows a black square that represents aerosol contaminated by some compound as it would be measured by a “smoking machine” and extrapolated to dosage from vaping in one day. This black square is located inside the white square that represents total uncontaminated air that is inhaled in a day by a vaper. The relative sizes of the two squares are exaggerated as the volume of aerosol generated in vaping relative to inhaled air is much smaller than is illustrated in the figure. *Panel ****B ***shows how exposure from contaminated air (black dots) is diluted over a day for appropriate comparison to occupational exposure limits that are expressed in terms of “time-weighted average” or average contamination over time rather than as instantaneous exposures. Exposure during vaping occurs in a dynamic process where the atmosphere inhaled by the vaper alternates between the smaller black and larger white squares in *Panel ****A***. Thus, the concentration of contaminants that a vaper is exposed to over a day is much smaller than that which is measured in the aerosol (and routinely improperly cited as reason for concern about “high” exposures).

It was also unclear from [38] what the volume of aerosol sampled was – a critical item for extrapolating to personal exposure and a common point of ambiguity in the published reports. However, in a personal exchange with the authors of [38] [July 11, 2013], it was clarified that the sampling pump drew air at 500 mL/min through e-cigarette for 10 min, allowing more appropriate calculations for estimation of health risk that are presented below. Such misleading reporting is common in the field that confuses concentration in the aerosol (typically measured directly) with concentration in the air inhaled by the vaper (never determined directly and currently requiring additional assumptions and modeling). This is important because the volume of aerosol inhaled (maximum ~8 L/day) is small compared to the volume of air inhaled daily (8 L/min); this point is illustrated in the Figure 1.

A similar but more extreme consideration applies to the exposure of bystanders which is almost certainly several orders of magnitude lower than the exposure of vapers. In part this is due to the absorption, rather than exhalation, of a portion of the aerosol by the vapers: there is no equivalent to the “side-stream” component of exposure to conventional cigarettes, so all of the exposure to a bystander results from exhalation. Furthermore, any environmental contamination that results from exhalation of aerosol by vaper will be diluted into the air prior to entering a bystander’s personal breathing zone. Lastly, the number of puffs that affect exposure to bystander is likely to be much smaller than that of a vaper unless we are to assume that vaper and bystander are inseparable.

It is unhelpful to report the results in cigarette-equivalents in assessments that are not about cigarette exposure, as in [43], because this does not enable one to estimate exposures of vapers. To be useful for risk assessment, the results on the chemistry of the aerosols and liquids must be reported in a form that enables the calculations in Equations 1 and 2. It must be also be noted that typical investigations consisted of qualitative and quantitative phases such that quantitative data is available mostly on compounds that passed the qualitative screen. In the qualitative phase, presence of the compounds above a certain limit of detection is determined. In the quantitative phase, the amount of only the compounds that are detected in the qualitative phase is estimated. This biased all reports on concentration of compounds towards both higher levels and chemicals which a particular lab was most adept at analyzing.

### Declared Ingredients: comparison to occupational exposure limits

#### ***Propylene glycol and glycerin***

Propylene glycol and glycerin have the default or precautionary 8-hour TLV of 10 mg/m^3^ set for all organic mists with no specific exposure limits or identified toxicity (http://www.osha.gov/dts/chemicalsampling/data/CH_243600.html; accessed July 5, 2013). These interim TLVs tend to err on the side of being too high and are typically lowered if evidence of harm to health accumulates. For example, in a study that related exposure of theatrical fogs (containing propylene glycol) to respiratory symptoms [46], “mean personal inhalable aerosol concentrations were 0.70 mg/m^3^ (range 0.02 to 4.1)” [47]. The only available estimate of propylene concentration of propylene glycol in the aerosol indicates personal exposure on the order of 3–4 mg/m^3^ in the personal breathing zone over 8 hours (under the assumptions we made for all other comparisons to TLVs) [2]. The latest (2006) review of risks of occupational exposure to propylene glycol performed by the Health Council of the Netherlands (known for OELs that are the most protective that evidence supports and based exclusively on scientific considerations rather than also accounting for feasibility as is the case for the TLVs) recommended exposure limit of 50 mg/m^3^ over 8 hours; concern over short-term respiratory effects was noted [http://www.gezondheidsraad.nl/sites/default/files/200702OSH.pdf; accessed July 29, 2013]. Assuming extreme consumption of the liquid per day via vaping (5 to 25 ml/day and 50-95% propylene glycol in the liquid),^b^ levels of propylene glycol in inhaled air can reach 1–6 mg/m^3^. It has been suggested that propylene glycol is very rapidly absorbed during inhalation [4,6] making the calculation under worst case scenario of all propylene glycol becoming available for inhalation credible. It must also be noted that when consuming low-nicotine or nicotine-free liquids, the chance to consume larger volumes of liquid increases (large volumes are needed to reach the target dose or there is no nicotine feedback), leading to the upper end of propylene glycol and glycerin exposure. Thus, estimated levels of exposure to propylene glycol and glycerin are close enough to TLV to warrant concern. However, it is also important to consider that propylene glycol is certainly not all absorbed because visible aerosol is exhaled in typical vaping. Therefore, the current calculation is in the spirit of a worst case assumption that is adopted throughout the paper.

#### ***Nicotine***

Nicotine is present in most e-cigarette liquids and has TLV of 0.5 mg/m^3^ for average exposure intensity over 8 hours. If approximately 4 m^3^ of air is inhaled in 8 hours, the consumption of 2 mg nicotine from e-cigarettes in 8 hours would place the vaper at the occupational exposure limit. For a liquid that contains 18 mg nicotine/ml, TLV would be reached upon vaping ~0.1-0.2 ml of liquid in a day, and so is achieved for most anyone vaping nicotine-containing e-cigarettes [1]. Results presented in [25] on 16 e-cigarettes also argue in favor of exceedance of TLV from most any nicotine-containing e-cigarette, as they predict >2 mg of nicotine released to aerosol in 150 puffs (daily consumption figure adopted in this report). But as noted above, since delivery of nicotine is the purpose of nicotine-containing e-cigarettes, the comparison to limits on unintended, unwanted exposures does not suggest a problem and serves merely to offer complete context. If nicotine is present but the liquid is labeled as zero-nicotine [25,44], it could be treated as a contaminant, with the vaper not intending to consume nicotine and the TLV, which would be most likely exceeded, is relevant. However, when nicotine content is disclosed, even if inaccurately, then comparison to TLV is not valid. Accuracy in nicotine content is a concern with respect to truth in advertising rather than unintentional exposure, due to presumed (though not yet tested) self-regulation of consumption by persons who use e-cigarettes as a source of nicotine.

Overall, the declared ingredients in the liquid would warrant a concern by standards used in occupational hygiene, provided that comparison to occupational exposure limits is valid, as discussed in the introduction. However, this is not to say that the exposure is affirmatively believed to be harmful; as noted, the TLVs for propylene glycol and glycerin mists is based on uncertainty rather than knowledge. These TLVs are not derived from knowledge of toxicity of propylene glycol and glycerin mists, but merely apply to any compound of no known toxicity present in workplace atmosphere. This aspect of the exposure from e-cigarettes simply has little precedent (but see study of theatrical fogs below). Therefore, the exposure will provide the first substantial collection evidence about the effects, which calls for monitoring of both exposure levels and outcomes, even though there are currently no grounds to be concerned about the immediate or chronic health effects of the exposure. The argument about nicotine is presented here for the sake of completeness and consistency of comparison to TLVs, but in itself does not affect the conclusions of this analysis because it should not be modeled as if it were a contaminant when declared as an ingredient in the liquid.

### Contaminants

#### ***Polycyclic aromatic hydrocarbons***

Polycyclic aromatic hydrocarbons (PAH) were quantified in several reports in aerosols [5,6,43] and liquids [7,19,42]. These compounds include well-known carcinogens, the levels of which are not subject to TLV but are instead to be kept “as low as reasonably achievable” [10]. For PAH, only non-carcinogenic pyrene that is abundant in the general environment was detected at 36 ng/cartridge in 5 samples of liquid [7]; PAHs were not detected in most of the analyses of aerosols, except for chrysene in the analysis of the aerosol of one e-cigarette [43].

#### ***Tobacco-specific nitrosamines***

The same risk assessment considerations that exist for PAH also hold for carcinogenic tobacco-specific nitrosamines (TSNAs) [48] for which no occupational exposure limits exist because (a) these exposures do not appear to occur in occupational settings often enough to warrant development of TLVs, and (b) it is currently accepted in establishing TLVs that carcinogens do not have minimal thresholds of toxicity. As expected, because the TSNAs are contaminants of nicotine from tobacco leaf, there is also evidence of association between nicotine content of the liquid and TSNA concentrations, with reported concentrations <5 ng/cartridge tested [7]. Smaller studies of TSNA content in liquids are variable, with some not reporting any detectable levels [18,33,35] and others clearly identifying these compounds in the liquids when controlling for background contamination (n = 9) [23]. Analyses of aerosols indicate that TSNAs are present in amounts that can results in doses of < ng/day [5,33] to μg/day [8] (assuming 150 puffs/day) (see also [43]). The most comprehensive survey of TSNA content of 105 samples of liquids from 11 manufactures indicates that almost all tested liquids (>90%) contained TSNAs in μg/L quantities [36]. This is roughly equivalent to 1/1000 of the concentration of TSNAs in modern smokeless tobacco products (like snus), which are in the ppm range [48]. For example, 10 μg/L (0.01 ppm) of total TSNA in liquid [36] can translate to a daily dose of 0.025–0.05 μg from vaping (worst case assumption of 5 ml liquid/day); if 15 g of snus is consumed a day [49] with 1 ppm of TSNAs [48] and half of it were absorbed, then the daily dose is estimated to be 7.5 μg, which is 150–300 times that due to the worst case of exposure from vaping. Various assumptions about absorption of TSNAs alter the result of this calculation by a factor that is dwarfed in magnitude compared to that arising from differences considered above. This is reassuring because smokeless tobacco products, such as snus, pose negligible cancer risk [50], certainly orders of magnitude smaller than smoking (if one considers the chemistry of the products alone). In general, it appears that the cautious approach in face of variability and paucity of data is to seek better understanding of the predictors of presence of TSNA in liquids and aerosols so that measures for minimizing exposure to TSNAs from aerosols can be devised. This can include considering better control by manufactures who extract the nicotine from tobacco leaf.

#### ***Volatile organic compounds***

Total volatile organic compounds (VOC) were determined in aerosol to be non-detectable [3] except in one sample that appeared to barely exceed the background concentration of 1 mg/m^3^ by 0.73 mg/m^3^[6]. These results are corroborated by analyses of liquids [19] and most likely testify to insensitivity of employed analytic methods for total VOC for characterizing aerosol generated by e-cigarettes, because there is ample evidence that specific VOC are present in the liquids and aerosols.^c^ Information on specific commonly detected VOC in the aerosol is given in Table 1. It must be observed that these reported concentrations are for analyses that first observed qualitative evidence of the presence of a given VOC and thus represent worst case scenarios of exposure when VOC is present (i.e. zero-level exposures are missing from the overall summary of worst case exposures presented here). For most VOC and aldehydes, one can predict the concentration in air inhaled by a vaper to be < <1% of TLV. The only exceptions to this generalization are:

(a) acrolein: ~1% of TLV (average of 12 measurements) [40] and measurements at a mean of 2% of TLV (average of 150 measurements) [41] and

(b) formaldehyde: between 0 and 3% of TLV based on 18 tests (average of 12 measurements at 2% of TLV, the most reliable test) [40] and an average of 150 results at 4% of TLV [41].

**Table 1:** Exposure predictions based on analysis of aerosols generated by smoking machines: volatile organic compounds

**Compound**	**N**^**#**^	**Estimated concentration in personal breathing zone**		**Ratio of most stringent TLV (%)**		**Reference**
		**PPM**	**mg/m**^**3**^	**Calculated directly**	**Safety factor 10**	
Acetaldehyde	1	0.005		0.02	0.2	[5]
	3	0.003		0.01	0.1	[4]
	12	0.001		0.004	0.04	[8]
	1	0.00004		0.0001	0.001	[3]
	1	0.0002		0.001	0.008	[3]
	150	0.001		0.004	0.04	[40,41]
	1	0.008		0.03	3	[38]
Acetone	1	0.002		0.0003	0.003	[38]
	150	0.0004		0.0001	0.001	[40,41]
Acrolein	12	0.001		1	13	[8]
	150	0.002		2	20	[40,41]
	1	0.006		6	60	[38]
Butanal	150	0.0002		0.001	0.01	[40,41]
Crotonaldehyde	150		0.0004	0.01	0.1	[40,41]
Formaldehyde	1	0.002		0.6	6	[5]
	3	0.008		3	30	[4]
	12	0.006		2	20	[8]
	1	<0.0003		<0.1	<1	[3]
	1	0.0003		0.1	1	[3]
	150	0.01		4	40	[40,41]
	1	0.009		3	30	[38]
Glyoxal	1		0.002	2	20	[38]
	150		0.006	6	60	[40,41]
o-Methylbenzaldehyde	12		0.001	0.05	0.5	[8]
p,m-Xylene	12		0.00003	0.001	0.01	[8]
Propanal	3	0.002		0.01	0.1	[4]
	150	0.0006		0.002	0.02	[40,41]
	1	0.005		0.02	0.2	[38]
Toluene	12	0.0001		0.003	0.03	[8]
Valeraldehyde	150		0.0001	0.0001	0.001	[40,41]

^#^Average is presented when N > 1.

Levels of acrolein in exhaled aerosol reported in [6] were below 0.0016 mg/m^3^ and correspond to predicted exposure of <1% of TLV (Table 2). It must re-emphasized that all calculations based on one electronic cigarette analyzed in [38] are best treated as qualitative in nature (i.e. indicating presence of a compound without any particular meaning attached to the reported level with respect to typical levels) due to great uncertainty about whether the manner in which the e-cigarette was operated could have resulted in overheating that led to generation of acrolein in the aerosol. In fact, a presentation made by the author of [38] clearly stated that the “atomizer, generating high concentration carbonyls, had been burned black” [40,41]. In unpublished work, [40] there are individual values of formaldehyde, acrolein and glyoxal that approach TLV, but it is uncertain how typical these are because there is reason to believe the liquid was overheated; considerable variability among brands of electronic cigarettes was also noted. Formaldehyde and other aldehydes, but not acrolein, were detected in the analysis one e-cigarette [43]. The overwhelming majority of the exposure to specific VOC that are predicted to result from inhalation of the aerosols lie far below action level of 50% of TLV at which exposure has to be mitigated according to current code of best practice in occupational hygiene [51].

**Table 2:** Exposure predictions for volatile organic compounds based on analysis of aerosols generated by volunteer vapers

**Compound**	**N**^**#**^	**Estimated concentration in personal breathing zone (ppm)**	**Ratio of most stringent TLV (%)**		**Reference**
			**Calculated directly**	**Safety factor 10**	
2-butanone (MEK)	3	0.04	0.02	0.2	[4]
	1	0.002	0.0007	0.007	[6]
2-furaldehyde	3	0.01	0.7	7	[4]
Acetaldehyde	3	0.07	0.3	3	[4]
Acetic acid	3	0.3	3	30	[4]
Acetone	3	0.4	0.2	2	[4]
Acrolein	1	<0.001	<0.7	<7	[6]
Benzene	3	0.02	3	33	[4]
Butyl hydroxyl toluene	1	4E-05	0.0002	0.002	[6]
Isoprene	3	0.1	7	70	[4]
Limonene	3	0.009	0.03	0.3	[4]
	1	2E-05	0.000001	0.00001	[6]
m,p-Xyelen	3	0.01	0.01	0.1	[4]
Phenol	3	0.01	0.3	3	[4]
Propanal	3	0.004	0.01	0.1	[4]
Toluene	3	0.01	0.07	0.7	[4]

^#^Average is presented when N > 1.

Finding of an unusually high level of formaldehyde by Schripp *et al.*[4] – 0.5 ppm predicted vs. 15-minute TLV of 0.3 ppm (not given in Table 2) – is clearly attributable to endogenous production of formaldehyde by the volunteer smoker who was consuming e-cigarettes in the experimental chamber, since there was evidence of build-up of formaldehyde prior to vaping and liquids used in the experiments did not generate aerosol with detectable formaldehyde. This places generalizability of other findings from [4] in doubt, especially given that the only other study of exhaled air by vapers who were not current smokers reports much lower concentrations for the same compounds [6] (Table 2). It should be noted that the report by Romagna *et al.*[6] employed more robust methodology, using 5 volunteer vapers (no smokers) over an extended period of time. Except for benzene, acetic acid and isoprene, all calculated concentrations for detected VOC were much below 1% of TLV in exhaled air [6]. In summary, these results do not indicate that VOC generated by vaping are of concern by standards used in occupational hygiene.

Diethylene glycol and ethylene glycol became a concern following the report of their detection by FDA [44], but these compounds are not detected in the majority of tests performed to date [3,15,17,19,23]. Ten batches of the liquid tested by their manufacture did not report any diethylene glycol above 0.05% of the liquid [42]. Methods used to detect diethylene glycol appear to be adequate to be informative and capable of detecting the compound in quantities < <1% of TLV [15,17,23]. Comparison to TLV is based on a worst case calculation analogous to the one performed for propylene glycol. For diethylene glycol, TLV of 10 mg/m^3^ is applicable (as in the case of all aerosols with no know toxicity by inhalation), and there is a recent review of regulations of this compound conducted for the Dutch government by the Health Council of the Netherlands (jurisdiction with some of the most strict occupational exposure limits) that recommended OEL of 70 mg/m^3^ and noted lack of evidence for toxicity following inhalation [http://www.gezondheidsraad.nl/sites/default/files/200703OSH.pdf; accessed July 29; 2013]. In conclusion, even the quantities detected in the single FDA result were of little concern, amounting to less than 1% of TLV.

#### ***Inorganic compounds***

Special attention has to be paid to the chemical form of compounds when there is detection of metals and other elements by inductively coupled plasma mass spectrometry (ICP-MS) [8,26]. Because the parent molecule that occurs in the aerosol is destroyed in such analysis, the results can be misleading and not interpretable for risk assessment. For example, the presence of sodium (4.18 μg/10 puffs) [26] does not mean that highly reactive and toxic sodium metal is in the aerosol, which would be impossible given its reactivity, but most likely means the presence of the ubiquitous compound that contains sodium, dissolved table salt (NaCl). If so, the corresponding daily dose of NaCl that arises from these concentrations from 150 puffs is about 10,000 times lower than allowable daily intake according to CDC (http://www.cdc.gov/features/dssodium/; accessed July 4, 2013). Likewise, a result for presence of silica is meaningless for health assessment unless the crystalline form of SiO_2_ is known to be present. When such ambiguity exists, a TLV equivalence calculation was not performed. We compared concentrations to TLVs when it was even remotely plausible that parent molecules were present in the aqueous solution. However, even these are to be given credence only in an extremely pessimistic analyst, and further investigation by more appropriate analytical methods could clarify exactly what compounds are present, but is not a priority for risk assessment.

It should also be noted that one study that attempted to quantify metals in the liquid found none above 0.1-0.2 ppm levels [7] or above unspecified threshold [19]. Table 3 indicates that most metals that were detected were present at <1% of TLV even if we assume that the analytical results imply the presence of the most hazardous molecules containing these elements that can occur in aqueous solution. For example, when elemental chromium was measured, it is compared to TLV for insoluble chromium IV that has the lowest TLV of all chromium compounds. Analyses of metals given in [43] are not summarized here because of difficulty with translating reported units into meaningful terms for comparison with the TLV, but only mercury (again with no information on parent organic compound) was detected in trace quantities, while arsenic, beryllium, chromium, cadmium, lead and nickel were not. Taken as the whole, it can be inferred that there is no evidence of contamination of the aerosol with metals that warrants a health concern.

**Table 3:** **Exposure predictions based on analysis of aerosols generated by smoking machines: inorganic compounds**^**#**^

**Element quantified**	**Assumed compound containing the element for comparison with TLV**	**N**^**##**^	**Estimated concentration in personal breathing zone (mg/m**^**3**^**)**	**Ratio of most stringent TLV (%)**		**Reference**
				**Calculated directly**	**Safety factor 10**	
Aluminum	Respirable Al metal & insoluble compounds	1	0.002	0.2	1.5	[26]
Barium	Ba & insoluble compounds	1	0.00005	0.01	0.1	[26]
Boron	Boron oxide	1	0.02	0.1	1.5	[26]
Cadmium	Respirable Cd & compounds	12	0.00002	1	10	[8]
Chromium	Insoluble Cr (IV) compounds	1	3E-05	0.3	3	[26]
Copper	Cu fume	1	0.0008	0.4	4.0	[26]
Iron	Soluble iron salts, as Fe	1	0.002	0.02	0.2	[26]
Lead	Inorganic compounds as Pb	1	7E-05	0.1	1	[26]
		12	0.000025	0.05	0.5	[8]
Magnesium	Inhalable magnesium oxide	1	0.00026	0.003	0.03	[26]
Manganese	Inorganic compounds, as Mn	1	8E-06	0.04	0.4	[26]
Nickel	Inhalable soluble inorganic compounds, as Ni	1	2E-05	0.02	0.2	[26]
		12	0.00005	0.05	0.5	[8]
Potassium	KOH	1	0.001	0.1	1	[26]
Tin	Organic compounds, as Sn	1	0.0001	0.1	1	[26]
Zinc	Zinc chloride fume	1	0.0004	0.04	0.4	[26]
Zirconium	Zr and compounds	1	3E-05	0.001	0.01	[26]
Sulfur	SO_2_	1	0.002	0.3	3	[26]

^#^The actual molecular form in the aerosol unknown and so worst case assumption was made if it was physically possible (e.g. it is not possible for elemental lithium & sodium to be present in the aerosol); there is no evidence from the research that suggests the metals were in the particular highest risk form, and in most cases a general knowledge of chemistry strongly suggests that this is unlikely. Thus, the TLV ratios reported here probably do not represent the (much lower) levels that would result if we knew the molecular forms.

^##^Average is presented when N > 1.

### Consideration of exposure to a mixture of contaminants

All calculations conducted so far assumed only one contaminant present in clean air at a time. What are the implications of small quantities of various compounds with different toxicities entering the personal breathing zone at the same time? For evaluation of compliance with exposure limits for mixtures, Equation 3 is used:

(3) $${\mathrm{OEL}}_{\text{mixture}}=\sum_{i-1}^{n}\left(C_{i}/\mathrm{TL}V_{i}\right),$$

where C_*i*_ is the concentration of the *i*^th^ compound (*i* = 1,…,*n*, where n > 1 is the number of ingredients present in a mixture) in the contaminated air and TLV_*i*_ is the TLV for the *i*^th^ compound in the contaminated air; if OEL_mixture_ > 1, then there is evidence of the mixture exceeding TLV.

The examined reports detected no more than 5–10 compounds in the aerosol, and the above calculation does not place any of them out of compliance with TLV for mixture. Let us imagine that 50 compounds with TLVs were detected. Given that the aerosol tends to contain various compounds at levels, on average, of no more than 0.5% of TLV (Tables 1 and 3), such a mixture with 50 ingredients would be at 25% of TLV, a level that is below that which warrants a concern, since the “action level” for implementation of controls is traditionally set at 50% of TLV to ensure that the majority of persons exposed have personal exposure below mandated limit [51]. Pellerino *et al.*[2] reached conclusions similar to this review based on their single experiment: contaminants in the liquids that warrant health concerns were present in concentrations that were less than 0.1% of that allowed by law in the European Union. Of course, if the levels of the declared ingredients (propylene glycol, glycerin, and nicotine) are considered, the action level would be met, since those ingredients are present in the concentrations that are near the action level. There are no known synergistic actions of the examined mixtures, so Equation 3 is therefore applicable. Moreover, there is currently no reason to suspect that the trace amounts of the contaminants will react to create compounds that would be of concern.

## Conclusions

By the standards of occupational hygiene, current data do not indicate that exposures to vapers from contaminants in electronic cigarettes warrant a concern. There are no known toxicological synergies among compounds in the aerosol, and mixture of the contaminants does not pose a risk to health. However, exposure of vapers to propylene glycol and glycerin reaches the levels at which, if one were considering the exposure in connection with a workplace setting, it would be prudent to scrutinize the health of exposed individuals and examine how exposures could be reduced. This is the basis for the recommendation to monitor levels and effects of prolonged exposure to propylene glycol and glycerin that comprise the bulk of emissions from electronic cigarettes other than nicotine and water vapor. From this perspective, and taking the analogy of work on theatrical fogs [46,47], it can be speculated that respiratory functions and symptoms (but not cancer of respiratory tract or non-malignant respiratory disease) of the vaper is of primary interest. Monitoring upper airway irritation of vapers and experiences of unpleasant smell would also provide early warning of exposure to compounds like acrolein because of known immediate effects of elevated exposures (http://www.atsdr.cdc.gov/toxprofiles/tp124-c3.pdf; accessed July 11, 2013). However, it is questionable how much concern should be associated with observed concentrations of acrolein and formaldehyde in the aerosol. Given highly variable assessments, closer scrutiny is probably warranted to understand sources of this variability, although there is no need at present to be alarmed about exceeding even the occupational exposure limits, since occurrence of occasional high values is accounted for in established TLVs. An important clue towards a productive direction for such work is the results reported in [40,41] that convincingly demonstrate how heating the liquid to high temperatures generates compounds like acrolein and formaldehyde in the aerosol. A better understanding about the sources of TSNA in the aerosol may be of some interest as well, but all results to date consistently indicate quantities that are of no more concern than TSNA in smokeless tobacco or nicotine replacement therapy (NRT) products. Exposures to nicotine from electronic cigarettes is not expected to exceed that from smoking due to self-titration [11]; it is only a concern when a vaper does not intend to consume nicotine, a situation that can arise from incorrect labeling of liquids [25,44].

The cautions about propylene glycol and glycerin apply only to the exposure experienced by the vapers themselves. Exposure of bystanders to the listed ingredients, let alone the contaminants, does not warrant a concern as the exposure is likely to be orders of magnitude lower than exposure experienced by vapers. Further research employing realistic conditions could help quantify the quantity of exhaled aerosol and its behavior in the environment under realistic worst-case scenarios (i.e., not small sealed chambers), but this is not a priority since the exposure experienced by bystanders is clearly very low compared to the exposure of vapers, and thus there is no reason to expect it would have any health effects.

The key to making the best possible effort to ensure that hazardous exposures from contaminants do not occur is ongoing monitoring of actual exposures and estimation of potential ones. Direct measurement of personal exposures is not possible in vaping due to the fact the aerosol is inhaled directly, unless, of course, suitable biomarkers of exposure can be developed. The current review did not identify any suitable biomarkers, though cotinine is a useful proxy for exposure to nicotine-containing liquids. Monitoring of potential composition of exposures is perhaps best achieved though analysis of aerosol generated in a manner that approximates vaping, for which better insights are needed on how to modify “smoking machines” to mimic vaping given that there are documented differences in inhalation patterns [52] that depend on features of e-cigarettes [14]. These smoking machines would have to be operated under a realistic mode of operation of the atomizer to ensure that the process for generation of contaminants is studied under realistic temperatures. To estimate dosage (or exposure in personal breathing zone), information on the chemistry of the aerosol has to be combined with models of the inhalation pattern of vapers, mode of operation of e-cigarettes and quantities of liquid consumed. Assessment of exhaled aerosol appears to be of little use in evaluating risk to vapers due to evidence of qualitative differences in the chemistry of exhaled and inhaled aerosol.

Monitoring of liquid chemistry is easier and cheaper than assessment of aerosols. This can be done systematically as a routine quality control measure by the manufacturers to ensure uniform quality of all production batches. However, we do not know how this relates to aerosol chemistry because previous researchers did not appropriately pair analyses of chemistry of liquids and aerosols. It is standard practice in occupational hygiene to analyze the chemistry of materials generating an exposure, and it is advisable that future studies of the aerosols explicitly pair these analyses with examination of composition of the liquids used to generate the aerosols. Such an approach can lead to the development of predictive models that relate the composition of the aerosol to the chemistry of liquids, the e-cigarette hardware, and the behavior of the vaper, as these, if accurate, can anticipate hazardous exposures before they occur. The current attempt to use available data to develop such relationships was not successful due to studies failing to collect appropriate data. Systematic monitoring of quality of the liquids would also help reassure consumers and is best done by independent laboratories rather than manufactures to remove concerns about impartiality (real or perceived).

Future work in this area would greatly benefit from standardizing laboratory protocols (e.g. methods of extraction of compounds from aerosols and liquids, establishment of “core” compounds that have to be quantified in each analysis (as is done for PAH and metals), development of minimally informative detection limits that are needed for risk assessment, standardization of operation of “vaping machine”, etc.), quality control experiments (e.g. suitable positive and negative controls without comparison to conventional cigarettes, internal standards, estimation of % recovery, etc.), and reporting practices (e.g. in units that can be used to estimate personal exposure, use of uniform definitions of limits of detection and quantification, etc.), all of which would improve on the currently disjointed literature. Detailed recommendations on standardization of such protocols lie outside of scope of this report.

All calculations conducted in this analysis are based on information about patterns of vaping and the content of aerosols and liquids that are highly uncertain in their applicability to “typical” vaping as it is currently practiced and says even less about future exposures due to vaping (e.g. due to development of new technology). However, this is similar to assessments that are routinely performed in occupational hygiene for novel technology as it relied on “worst case” calculations and safety margins that attempt to account for exposure variability. The approach adopted here and informed by some data is certainly superior to some currently accepted practices in the regulatory framework in occupational health that rely purely on description of emission processes to make claims about potential for exposure (e.g. [53]). Clearly, routine monitoring of potential and actual exposure is required if we were to apply the principles of occupational hygiene to vaping. Detailed suggestions on how to design such exposure surveillance are available in [54].

While vaping is obvious not an occupational exposure, occupational exposure standards are the best available option to use. If there were a standard for voluntary consumer exposure to aerosols, it would be a better fit, but no such standard exists. The only candidate standard is the occupational standard, which is conservative (more protective) when considered in the context of voluntary exposures, as argued above, and any suggestion that another standard be used needs to be concrete and justified.

In summary, analysis of the current state of knowledge about the chemistry of contaminants in liquids and aerosols associated with electronic cigarettes indicates that there is no evidence that vaping produces inhalable exposures to these contaminants at a level that would prompt measures to reduce exposure by the standards that are used to ensure safety of workplaces. Indeed, there is sufficient evidence to be reassured that there are no such risks from the broad range of the studied products, though the lack of quality control standards means that this cannot be assured for all products on the market. However, aerosol generated during vaping on the whole, when considering the declared ingredients themselves, if it were treated in the same manner as an emission from industrial process, creates personal exposures that would justify surveillance of exposures and health among exposed persons. Due to the uncertainty about the effects of these quantities of propylene glycol and glycerin, this conclusion holds after setting aside concerns about health effects of nicotine. This conclusion holds notwithstanding the benefits of tobacco harm reduction, since there is value in understanding and possibly mitigating risks even when they are known to be far lower than smoking. It must be noted that the proposal for such scrutiny of “total aerosol” is not based on specific health concerns suggested by compounds that resulted in exceedance of occupational exposure limits, but is instead a conservative posture in the face of unknown consequences of inhalation of appreciable quantities of organic compounds that may or may not be harmful at doses that occur during vaping.

## Key conclusions

• Even when compared to workplace standards for involuntary exposures, and using several conservative (erring on the side of caution) assumptions, the exposures from using e-cigarettes fall well below the threshold for concern for compounds with known toxicity. That is, even ignoring the benefits of e-cigarette use and the fact that the exposure is actively chosen, and even comparing to the levels that are considered unacceptable to people who are not benefiting from the exposure and do not want it, the exposures would not generate concern or call for remedial action.

• Expressed concerns about nicotine only apply to vapers who do not wish to consume it; a voluntary (indeed, intentional) exposure is very different from a contaminant.

• There is no serious concern about the contaminants such as volatile organic compounds (formaldehyde, acrolein, etc.) in the liquid or produced by heating. While these contaminants are present, they have been detected at problematic levels only in a few studies that apparently were based on unrealistic levels of heating.

• The frequently stated concern about contamination of the liquid by a nontrivial quantity of ethylene glycol or diethylene glycol remains based on a single sample of an early-technology product (and even this did not rise to the level of health concern) and has not been replicated.

• Tobacco-specific nitrosamines (TSNA) are present in trace quantities and pose no more (likely much less) threat to health than TSNAs from modern smokeless tobacco products, which cause no measurable risk for cancer.

• Contamination by metals is shown to be at similarly trivial levels that pose no health risk, and the alarmist claims about such contamination are based on unrealistic assumptions about the molecular form of these elements.

• The existing literature tends to overestimate the exposures and exaggerate their implications. This is partially due to rhetoric, but also results from technical features. The most important is confusion of the concentration in aerosol, which on its own tells us little about risk to heath, with the relevant and much smaller total exposure to compounds in the aerosol averaged across all air inhaled in the course of a day. There is also clear bias in previous reports in favor of isolated instances of highest level of chemical detected across multiple studies, such that average exposure that can be calculated are higher than true value because they are “missing” all true zeros.

• Routine monitoring of liquid chemistry is easier and cheaper than assessment of aerosols. Combined with an understanding of how the chemistry of the liquid affects the chemistry of the aerosol and insights into behavior of vapers, this can serve as a useful tool to ensure the safety of e-cigarettes.

• The only unintentional exposures (i.e., not the nicotine) that seem to rise to the level that they are worth further research are the carrier chemicals themselves, propylene glycol and glycerin. This exposure is not known to cause health problems, but the magnitude of the exposure is novel and thus is at the levels for concern based on the lack of reassuring data.

### Endnotes

^a^Atmosphere that contains air inhaled by a person.

^b^This estimate of consumption was derived from informal reports from vaping community; 5 ml/day was identified as a high but not rare quantity of consumption and 25 ml/day was the high end of claimed use, though some skepticism was expressed about whether the latter quantity was truly possible. High-quality formal studies to verify these figures do not yet exist but they are consistent with report of Etter (2012).

^c^The term “VOC” loosely groups together all organic compounds present in aerosol and because the declared ingredients of aerosol are organic compounds, it follows that “VOC are present”.

## Competing interests

Funding for this work was provided by The Consumer Advocates for Smoke-free Alternatives Association (CASAA) Research Fund. CASAA is an all-volunteer, donation-funded, non-profit organization devoted to defending consumer access to and promoting tobacco harm reduction; it is a consumer (not industry) advocacy NGO. For more information, see http://casaa.org/. CASAA exercised no editorial control over the author’s writing or analysis: the author, not the funder, had full control of the content.

## Authors’ information

IB is trained in both occupational hygiene and epidemiology and thus is an expert in bring information that these two fields contribute to risk assessment and policy-making. IB does not and never has used any tobacco products. Current research was completed by him as independent research contract during otherwise unpaid summer months. IB is an Associate Professor at Drexel University and felt obliged to disclose his primary academic appointment but this work was completed outside of the structures of Drexel University.

## Pre-publication history

The pre-publication history for this paper can be accessed here:

http://www.biomedcentral.com/1471-2458/14/18/prepub

## Supplementary Material

Additional file 1 Summary of chemical analyses of e-cigarettes extracted from the literature. Click here for file

Additional file 2 **Key to identifying articles listed in *Additional file*** ***1***. Click here for file

Additional file 3 **Calculations conducted to compare reported results to threshold limit values.** Spreadsheet that implemented calculations summarized in the article. Click here for file
