# Supplementary material for: Peering through the mist: systematic review of what the chemistry of contaminants in electronic cigarettes tells us about health risks
Source: BMC Public Health. 2014 Jan 9;14:18. doi: 10.1186/1471-2458-14-18 (PMC3937158; doi:10.1186/1471-2458-14-18)
Supplement: Additional file 2 — Key to identifying articles listed in Additional file 1 . [file 1471-2458-14-18-S2.rtf]

Appendix B

References (in reverse order of RefID)

Murphy, J., Wong, E., & Lawton, M. 2010, Chemical and operational assessment of the Ruyan classic e-cigarette, British American Tobacco, Report P.474.
Ref ID: 1856
Scherer, G. 1999. Smoking behaviour and compensation: a review of the literature. Psychopharmacology (Berl), 145, (1) 1-20 available from: PM:10445368 
Ref ID: 1855
Benowitz, N.L. & Jacob, P., III 1984. Daily intake of nicotine during cigarette smoking. Clin.Pharmacol.Ther., 35, (4) 499-504 available from: PM:6705448 
Ref ID: 1854
eSmoke. Analytical reports on batches of e-liquids. http://www.esmoke.net/pages.php?pageid=20 . 2009. 7-11-2013. 
Ref Type: Electronic Citation
Ref ID: 1853
Uchiyama, S. <unpublished concentrations from experiments presented in https://www.jstage.jst.go.jp/article/bunsekikagaku/60/10/60_10_791/_pdf; through personal communications>.  2013. 
Ref Type: Unpublished Work
Ref ID: 1852
Ohta, K., Uchiyama, S., Inaba, Y., Nakagome, H., & Kunugita, N. 2011. Determination of carbonyl compounds generated from the electronic cigarette using coupled silica cartridges impregnated with hydroquinone and 2,4-dinitrophenylhydrazine. BUNSEKI KAGAKU, 60, (10) 791-797
Ref ID: 1851
Uchiyama, S., Inaba, Y., & Kunugita, N. 2010. Determination of acrolein and other carbonyls in cigarette smoke using coupled silica cartridges impregnated with hydroquinone and 2,4-dinitrophenylhydrazine. J.Chromatogr.A, 1217, (26) 4383-4388 available from: PM:20483418 
Ref ID: 1850
Tischer, M., Bredendiek-Kamper, S., Poppek, U., & Packroff, R. 2009. How safe is control banding? Integrated evaluation by comparing OELs with measurement data and using monte carlo simulation. Ann.Occup.Hyg., 53, (5) 449-462 available from: PM:19531808 
Ref ID: 1849
Digard, H., Errington, G., Richter, A., & McAdam, K. 2009. Patterns and behaviors of snus consumption in Sweden. Nicotine.Tob.Res., 11, (10) 1175-1181 available from: PM:19687306 
Ref ID: 1848
Holmes, J. R. 1994, How Much Air Do We Breathe?, California Environmental Protection Agency, Research Note 94-11.
Ref ID: 1847
Ganong, W.F. 1995. Review of medical physiology, 15 ed. London, Prentice Hall.
Ref ID: 1846
Kim, H.J. & Shin, H.S. 2013. Determination of tobacco-specific nitrosamines in replacement liquids of electronic cigarettes by liquid chromatography-tandem mass spectrometry. J.Chromatogr.A, 1291, 48-55 available from: PM:23602640 
Ref ID: 1845
Liedel, N. A., Busch, K. A., & Crouse, W. E. 1975, Exposure measurement action level and occupational environmental variability, US Departement of Health, Education, and Welfare, Public Health Service, Center for Disease Control, National Institute for Occupational Safety and Health, Division of Laboatories and Criteria Development, Cincinnati, OH, HEW Publication No. (NIOSH) 76-131.
Ref ID: 1844
The American Conference of Governmental Industrial Hygienists 2013. 2013 threshold limit values for chemical substances and physical agents & biological exposure indices Cincinnati, OH, ACGIH.
Ref ID: 1843
Etter, J.F. 2012. The Electronic Cigarette : an Alternative to Tobacco?  Jean-François Etter.
Ref ID: 1842
Varughese, S., Teschke, K., Brauer, M., Chow, Y., van, N.C., & Kennedy, S.M. 2005. Effects of theatrical smokes and fogs on respiratory health in the entertainment industry. Am.J.Ind.Med., 47, (5) 411-418 available from: PM:15828073 
Ref ID: 1841
Teschke, K., Chow, Y., van, N.C., Varughese, S., Kennedy, S.M., & Brauer, M. 2005. Exposures to atmospheric effects in the entertainment industry. J.Occup.Environ.Hyg., 2, (5) 277-284 available from: PM:15884183 
Ref ID: 1840
Trtchounian, A., Williams, M., & Talbot, P. 2010. Conventional and electronic cigarettes (e-cigarettes) have different smoking characteristics. Nicotine.Tob.Res., 12, (9) 905-912 available from: PM:20644205 
Ref ID: 1839
Trtchounian, A. & Talbot, P. 2011. Electronic nicotine delivery systems: is there a need for regulation? Tob.Control, 20, (1) 47-52 available from: PM:21139013 
Ref ID: 1838
Romagna, G. Clear Stream project: Citotoxicity assesment of an electronic cigarette vapour on 3T3 fibroblasts. Data review and comment. Updated data from the third laboratory analysis. http://clearstream.flavourart.it/site/wp-content/uploads/DATI/Clearstream - Updated data from the third laboratory analysis.pdf . 9-2-0013. 7-10-2013. 
Ref Type: Electronic Citation
Ref ID: 1837
Romagna, G., Zabarini, L., Barbiero, L., Boccietto, E., Todeschi, S., Caravati, E., Voster, D., & Farsalinos, K. 2012, Characterization of chemicals released to the environment by electronic cigarettes use (ClearStream-AIR project): is passive vaping a reality?, XIV Annual Meeting of the SRNT Europe 2012, Helsinki, Finland.
Ref ID: 1836
Anspach, T. 2011, Determination of tobacco-specific nitrosamines (TSNA) in aroma fluid for e-cigarettes, Eurofins Dr.Specht Laboratorien, 11-57021.
Ref ID: 1835
Sodoma, A. & Caggiano, C. M. 2007, Material characterization report, Analyze Inc., 0706.04.
Ref ID: 1834
Pattison, J. & Valenty, S. J. 2009, Material characterization report, Analyze Inc., 0910.14.
Ref ID: 1833
Lauterbach, J. H. & Laugesen, M. 2012, Comparison of toxicant levels in mainstream aerosols generated by Ruyan® electronic nicotine delivery systems(ENDS) and conventional cigarette products, In 51st Annual Meeting of the Society of Toxicology.
Ref ID: 1832
Graves, I. 2008, Report no. 468304. 60 ml sample of mist from 11 mg nicotine e-cigarette cartridge. Thermal desorption tubes., Hill Laboratories, Hamilton, New Zealand, 468304.
Ref ID: 1831
Farsalinos, K.E., Romagna, G., Tsiapras, D., Kyrzopoulos, S., & Voudris, V. 2013. Evaluation of electronic cigarette use (vaping) topography and estimation of liquid consumption: implications for research protocol standards definition and for public health authorities' regulation. Int.J Environ.Res.Public Health, 10, (6) 2500-2514 available from: PM:23778060 
Ref ID: 1830
Ingebrethsen, B.J., Cole, S.K., & Alderman, S.L. 2012. Electronic cigarette aerosol particle size distribution measurements. Inhal.Toxicol., 24, (14) 976-984 available from: PM:23216158 
Ref ID: 1829
Trehy, M.L., Ye, W., Hadwiger, M.E., Moore, T.W., Allgire, J.F., Woodruff, J.T., Ahadi, S.S., Black, J.C., & Westenberger, B.J. 2011. Analysis of Electronic Cigarette Cartridges, Refill Solutions, and Smoke for Nicotine and Nicotine Related Impurities. Journal of Liquid Chromatography & Related Technologies, 34, (14) 1442-1458 available from: ISI:000296230900012 
Ref ID: 1828
Goniewicz, M.L., Knysak, J., Gawron, M., Kosmider, L., Sobczak, A., Kurek, J., Prokopowicz, A., Jablonska-Czapla, M., Rosik-Dulewska, C., Havel, C., Jacob, P., III, & Benowitz, N. 2013. Levels of selected carcinogens and toxicants in vapour from electronic cigarettes. Tob.Control available from: PM:23467656 
Ref ID: 1827
Pellegrino, R.M., Tinghino, B., Mangiaracina, G., Marani, A., Vitali, M., Protano, C., Osborn, J.F., & Cattaruzza, M.S. 2012. Electronic cigarettes: an evaluation of exposure to chemicals and fine particulate matter (PM). Ann.Ig, 24, (4) 279-288 available from: PM:22913171 
Ref ID: 1826
Hecht, S.S. & Hoffmann, D. 1988. Tobacco-specific nitrosamines, an important group of carcinogens in tobacco and tobacco smoke. Carcinogenesis, 9, (6) 875-884 available from: PM:3286030 
Ref ID: 1825
Kubica, P., Kot-Wasik, A., Wasik, A., & Namiesnik, J. 2013. "Dilute & shoot" approach for rapid determination of trace amounts of nicotine in zero-level e-liquids by reversed phase liquid chromatography and hydrophilic interactions liquid chromatography coupled with tandem mass spectrometry-electrospray ionization. J Chromatogr.A, 1289, 13-18 available from: PM:23548207 
Ref ID: 1824
Gordon, S.M., Wallace, L.A., Brinkman, M.C., Callahan, P.J., & Kenny, D.V. 2002. Volatile organic compounds as breath biomarkers for active and passive smoking. Environmental Health Perspectives, 110, (7) 689-698 available from: PM:12117646 
Ref ID: 1823
Valance, C. & Ellicott, M. 2008, Analysis of chemical components from high, med & low nicotine cartridges; Report Number: D318.
Ref ID: 1822
Tytgat, J. 2007, "Super Smoker" expert report.
Ref ID: 1821
Wallace, L.A. & Pellizzari, E.D. 1995. Recent advances in measuring exhaled breath and estimating exposure and body burden for volatile organic compounds (VOCs). Environmental Health Perspectives, 103 Suppl 3, 95-98 available from: PM:7635121 
Ref ID: 1820
Fowles, J.R., Banton, M.I., & Pottenger, L.H. 2013. A toxicological review of the propylene glycols. Crit Rev.Toxicol., 43, (4) 363-390 available from: PM:23656560 
Ref ID: 1819
Laugesen, M. 2008, Safety report on the Ruyan® e-cigarette cartridge and inhaled aerosol .
Ref ID: 1818
Laugesen, M. Ruyan® E-cigarette bench-top tests . Society for Research on Nicotine and Tobacco, Dublin, April 30, 2009 . 2009. 
Ref Type: Abstract
Ref ID: 1817
Williams, M., Villarreal, A., Bozhilov, K., Lin, S., & Talbot, P. 2013. Metal and silicate particles including nanoparticles are present in electronic cigarette cartomizer fluid and aerosol. PLoS.One., 8, (3) e57987 available from: PM:23526962 
Ref ID: 1816
McAuley, T.R., Hopke, P.K., Zhao, J., & Babaian, S. 2012. Comparison of the effects of e-cigarette vapor and cigarette smoke on indoor air quality. Inhal.Toxicol., 24, (12) 850-857 available from: PM:23033998 
Ref ID: 1815
Warner, K.E. 2009. Tobacco research methodology: first things first. Cancer Epidemiol.Biomarkers Prev., 18, (12) 3140-3142 available from: PM:19959671 
Ref ID: 1814
Westenberger, B. J. 2009, Evaluation of e-cigarettes; DPATR-FY-09-23.
Ref ID: 1813
Ellicott, M. 2009, Analysis of components from "e-Juice XX HIGH 36mg/ml rated Nicotine Solution" ref S 55434; Report Number: E249A.
Ref ID: 1812
Cahn, Z. & Siegel, M. 2011. Electronic cigarettes as a harm reduction strategy for tobacco control: a step forward or a repeat of past mistakes? J Public Health Policy, 32, (1) 16-31 available from: PM:21150942 
Ref ID: 1811
Schripp, T., Markewitz, D., Uhde, E., & Salthammer, T. 2013. Does e-cigarette consumption cause passive vaping? Indoor.Air, 23, (1) 25-31 available from: PM:22672560 
Ref ID: 1810
Coulson, H. 2009, Analysis of components from Gamucci electronic cigarette cartridges, tobacco flavour regular smoking liquid; Report number: E98D.
Ref ID: 1809
Evans Analytical Group 2009, Gas chromatography mass spectroscopy(GC-MS) analysis report; JOB NUMBER C09Y8961.
Ref ID: 1808
eSmoking Institute. Identifying the concentration of chemical compounds and heavy metals in liquids. http://www.esmokinginstitute.com/en/node/32 . 2013. 
Ref Type: Electronic Citation
Ref ID: 1807
eSmoking Institute. Assessment of e-cigarette safety by comparing the chemical composition of e-cigarette aerosol and cigarette smoke from reference traditional cigarette. http://www.esmokinginstitute.com/en/node/31 . 2013. 
Ref Type: Electronic Citation
Ref ID: 1806
Alliance Technologies, L. 2009, Characterization of regal cartridges for electronic cigarettes - Phase II.
Ref ID: 1805
Alliance Technologies, L. 2009, Characterization of Regal cartridges for electronic cigarettes.
Ref ID: 1804
Alliance Technologies, L. 2009, Characterization of liquid "Smoke Juice" for electronic cigarettes.
Ref ID: 1803
Alliance Technologies, L. 2009, Chemical composition of "Instead" electronic cigarette smoke juice and vapor.
Ref ID: 1802
Goniewicz, M.L., Kuma, T., Gawron, M., Knysak, J., & Kosmider, L. 2013. Nicotine levels in electronic cigarettes. Nicotine.Tob.Res., 15, (1) 158-166 available from: PM:22529223 
Ref ID: 1801
Etter, J.F., Zather, E., & Svensson, S. 2013. Analysis of refill liquids for electronic cigarettes. Addiction available from: PM:23701634 
Ref ID: 1800
Etter, J.F., Bullen, C., Flouris, A.D., Laugesen, M., & Eissenberg, T. 2011. Electronic nicotine delivery systems: a research agenda. Tob.Control, 20, (3) 243-248 available from: PM:21415064 
Ref ID: 6
Hadwiger, M.E., Trehy, M.L., Ye, W., Moore, T., Allgire, J., & Westenberger, B. 2010. Identification of amino-tadalafil and rimonabant in electronic cigarette products using high pressure liquid chromatography with diode array and tandem mass spectrometric detection. J Chromatogr.A, 1217, (48) 7547-7555 available from: PM:20980012 
Ref ID: 1
